# Supplementary material for: Thermal remote sensing over heterogeneous urban and suburban landscapes using sensor-driven super-resolution
Source: PLoS One. 2022 Apr 6;17(4):e0266541. doi: 10.1371/journal.pone.0266541 (PMC8986004; doi:10.1371/journal.pone.0266541)
Supplement: Attachment — Submitted filename: R2103554-ResponseToReviewers-4_fix.docx [file pone.0266541.s001.docx]

*Reviewer #1: Dear authors:

I have found your research article quite interesting. I have carefully read your proposal. I have some questions and explanations I would like you to clarify for me. Your research tries to recover and deepen a method to improve the resolution of thermal images from a sensor point of view (sensor-driven), as opposed to other approaches (data-driven). The preceding methodology and your proposals for improvement are well defined. Except in the figures shown:
The order of the defined steps is not clear. In figure 1, there is a red arrow linking both steps of superresolution. Please clarify its meaning. In figure 2, MODIS B1,2(1Km) and (500 m) refer to one super-resolution step when they are images with different resolutions. The reader may be confused about the use of both images with different resolutions at the same super-resolution step.*
Thank you for your encouraging and constructive comments. Based on this and the comment from reviewer 2, we have added a new figure (Fig. 1) and explanation (Lines 124-130) to clarify the flowchart and corresponding steps. We also added a step-by-step explanation in Fig. 2 (Lines 131-149) and Fig. 3 (Line 178). For each super-resolution process, high-resolution images (*f*_high,k_), degraded high-resolution images (*f*_low,k_), and low-resolution images (*g*_low,k’_) are needed, and then super-resolution images (*g*_high,k’_) are output. For example, MODIS B1,2 (1 km) is *f*_low,k_, and B1,2 (500 m) is *f*_high,k_ in Fig. 3 (original Fig. 2).

*In line 103, (step 3) it is not clear what the meaning of "instant spectral library" is. I would thank the authors for clarifying this concept.*

We decided not to use this ambiguous term, but explicitly explain the concept (Line 112) as follows: “Step 3) Make a typical spectral pattern (i.e., correspondence between *f*_low,k_ and *g*_low,k’_) by clustering the homogeneous pixels within the entire study region.”

*In line 145, the authors refer to a super-resolution step using different bands simultaneously. The authors do not make clear how these many bands are handled. Clarification about how the bands fuse with the thermal image will be useful.*

Please see the abovementioned comments regarding the original Figs. 1 and 2, and the new Fig. 1. The super-resolution algorithm searches for the correspondence between *f*_low,k_ and *g*_low,k’_ from a neighboring pixel or the typical spectral pattern, and simultaneously positions *g*_high,k’_ for all bands k’.

*My major concern in this work, in the Results section, is about the statistics the authors use to determine the quality of the super-resolution transformation. RMSE is not a good index of image quality. It has been proved that very different images can have the same RMSE value. The same is true for the correlation coefficient (CC). There are other more appropriate image quality indices such as the ERGAS or the spectral mapping angle (SAM) for spectral reconstruction. If you want to analyze the spatial reconstruction, Peak Signal to Noise Ratio (PSNR) will measure the quality of this process. Please refer to those indices or justify the use of RMSE and CC properly.*

Based on this suggestion, we have added ERGAS and PSNR for accuracy assessment (Line 304; Table 2) and added new references [41, 42]. These indices also indicate a performance improvement with our algorithm, as was the case for the RMSE and CC. We did not use the SAM because we used the same ASTER reference data (band 14) for both MODIS bands (bands 31 and 32), and calculation of spectral similarity is not applicable in this case.

*Reviewer #2: The article presents a review of a methodology to obtain surface temperature values based on spatially resampled MODIS sensor data. In its presentation, the main objective of the article is to demonstrate the application of the method for urban and suburban areas, however the results and discussions presented do not respond to this expectation, which is the main point of the improvements that the article needs.*
Thank you for reviewing our manuscript. To demonstrate the results from the viewpoint of urban and suburban landscapes, we have added a new table to show the statistics related to the results (super-resolution thermal values and accuracy indices) for each land-use and land-cover type, including urban and six other categories (Table 4). This analysis, for example, quantitatively confirmed the heat island phenomenon in the urban region (Line 378), and revealed that the best super-resolution performance was observed in the urban region (Line 385). We have also added further findings of this analysis in the Discussion section (Line 378-397).

*In line 44, I suggest improving the presentation of the characteristics of the thermal images, perhaps presenting a table or in the text, detailing the resolutions of each sensor*

We have added a description of the degradation of the spatial resolution of thermal data with examples from MODIS and ASTER, in the Introduction (Line 36-40).

*In line 75, the authors seek to show the potential application of the algorithm, however the work does not show other results obtained with the use of the first algorithm that justify changes in it. I suggest that they present other works that analyzed the application of the 1 algorithm or made comparisons with other sensors*

Indeed, the applications on Mars [4] using THEMIS [27] (Line 69) and in urban and suburban areas [26] using ASTER (Line 77) were only the existing reports that we knew of, and neither provided sufficient quantitative accuracy assessment by comparison with independent validation data (Line 77). Therefore, this paper represents the first quantitative report of the performance of algorithm 1. We consider that this is a point of novelty of our research, and added an appropriate statement in the Introduction (Line 92).

*In line 87, In the final paragraph of the Introduction, I suggest adding the objectives and results expected by the work.*

We had already stated the research objective in the first sentence of the final paragraph (Line 80): “this work aims to investigate the potential applicability of the sensor-driven approach over a heterogeneous landscape, and to improve its primitive algorithm”. The expected result has been newly added (Line 92) based on this comment, relating to the abovementioned response to the original line 75.

*In line 103, in which he presents the routine for image treatment, I suggest improving the flowchart presented, using a number with stage indications, as this way the reader understands which steps the objective of the work changes and facilitates the replication of the work*

Based on this and the comment from reviewer 1, we have added a new figure to clarify the super-resolution process (Fig. 1), indicating the improved points (star symbols). A brief explanation of the overall process has also been added (Lines 124-130).

*In line 135, I suggest rescuing the reason for the proposed algorithm modification*

We have briefly stated the purpose of the refinement (Line 164): “To make the algorithm more straightforward and to create better radiometrically corrected results,”

*In line 144, Here again rescue the routine flowchart of the algorithm indicating where changes occurred and to present a new proposal*

We have added a description of how the refinements corresponded to each step (Line 166) by revisiting the flowchart as follows: “For each super-resolution process, refinement (1) concerns input-output correspondence and degraded image input, whereas refinement (2) concerns post-processing (both are indicated by a star symbol in the flowchart in Fig. 1).*”*

*In line 327, I recommend that the authors seek to contextualize the discussion of the results obtained by showing the temperature results according to the land uses present in the analyzed section. It is important and necessary to confirm statistically whether there were differences or changes in temperatures by observing the behavior of surface temperature in vegetated environments and urban spaces*

Please see the response to the general comment. We have added a statistical analysis regarding each land cover category in addition to a discussion (Lines 378-397).
