# Supplementary material for: Thermal remote sensing over heterogeneous urban and suburban landscapes using sensor-driven super-resolution
Source: PLoS One. 2022 Apr 6;17(4):e0266541. doi: 10.1371/journal.pone.0266541 (PMC8986004; doi:10.1371/journal.pone.0266541)
Supplement: Attachment — Submitted filename: R2103554-ResponseToReviewers-5.docx [file pone.0266541.s002.docx]

*Reviewer #1: I would like to thank the authors for their work in clarify my comments and suggestions about their original work. Now, in the new version, I have found this research paper clearer and the quality of the results are well established with the new statistical indices.*

*I am glad to propose your work for publishing.*

We are glad to hear your very positive comment, and thank you again for reviewing our paper.

*Reviewer #2: The authors met the requests made, presenting more details about how the workflow occurs for the first algorithm and presenting clarifications regarding the modifications and improvements proposed by the work that occurred in it.*

*It is still necessary to improve the statistical exploration of the results obtained, demonstrating the real improvement that the modification of the algorithm brings.*

*I suggest application of the non-parametric Wilcox test was applied, with 95% confidence, to determine if there was a significant difference between the radiance and brightness temperature occurring in different land covers and land uses for Algorithm1, 2 and 3.*

*This further analysis will enrich the results presented and effectively show the gains with the modification of the algorithm*

Thank you for your constructive comments. We understand that this suggestion refers to the statistical significance regarding improvement of the accuracy indices (PSNR and EAGAS) among the three algorithms, and we agree that the recommended analysis would be helpful for clarifying the gains due to modification of the algorithm. Therefore, we compared the accuracy indices for each category for the different algorithms (Table 5), and performed a Wilcoxon signed-rank test using classified samples (i.e., n = 7) as suggested. The results show that Algorithm 3 had a smaller ERGAS and a greater PSNR than Algorithm 2 with statistical significance (p < 0.01), and that Algorithm 2 had a smaller ERGAS and a greater PSNR than Algorithm 1 with statistical significance (p < 0.01). Therefore, for all categories, Algorithm 2 was better than Algorithm 1, and Algorithm 3 was better than both Algorithms 1 and 2 (Line 392). We added this description to the Discussion section.
